# The ICMJE *Recommendations* and pharmaceutical marketing – strengths, weaknesses and the unsolved problem of attribution in publication ethics

**DOI:** 10.1186/s12910-016-0103-7

**Published:** 2016-04-04

**Authors:** Alastair Matheson

**Affiliations:** Toronto, Canada

**Keywords:** ICMJE, Pharmaceutical industry, Marketing, Publishing industry, Publication ethics, Authorship, Contributorship, Data sharing, Conflict of interest, Bias, Attribution, Key opinion leader

## Abstract

**Background:**

The International Committee of Medical Journal Editors (ICMJE) *Recommendations* set ethical and editorial standards for article publication in most leading medical journals. Here, I examine the strengths and weaknesses of the *Recommendations* in the prevention of commercial bias in industry-financed journal literature, on three levels – scholarly discourse, article content, and article attribution.

**Discussion:**

With respect to overall discourse, the most important measures in the ICMJE *Recommendations* are for enforcing clinical trial registration and controlling duplicate publication. With respect to article content, the ICMJE promotes stringent author accountability and adherence to established reporting standards. However, the ICMJE accepts the use of commercial editorial teams to produce manuscripts, which is a potential source of bias, and accepts private company ownership and analysis of clinical trial data. New ICMJE guidance on data sharing will address but not eliminate problems of commercial data access. With respect to attribution, the *Recommendations* oppose guest authorship and encourage clear documentation of author contributions. However, they exclude writers from coauthorship; provide no specific advice on the attribution of commercial literature, for instance with respect to company authorship, author sequence or prominent commercial labeling; and endorse the use of fine print and euphemism. The ICMJE requires detailed author interest disclosures, but overlooks the interests of non-authors and companies, and does not recommend that interests most salient to the publication are highlighted. Together, these weaknesses facilitate “advocacy”-based marketing, in which literature planned, financed and produced by companies is fronted by academics, enabling commercial messages to be presented to customers by their respected clinical peers rather than companies themselves.

**Conclusions:**

The ICMJE *Recommendations* set important research and reporting standards, without which commercial bias would likely be a significantly greater problem than it is today. However, they also support practices of commercial data control, content development and attribution that run counter to science’s values of openness, objectivity and truthfulness. These weaknesses could be addressed with appropriate modifications to the *Recommendations*. The ICMJE should also disclose its own commercial interests and funding – not least because publishing organizations that finance it and pay the salaries of some member editors derive substantial revenues from industry.

## Background

Commercialization of academic medical literature is an important concern for journals, their readers and prescribing doctors. Good industry science should be welcomed into scholarly literature, but commercial bias may occur on several levels (Table 1). At the level of academic *discourse*, commercial decisions about which projects to undertake, data to publish, arguments to propose and articles to develop may distort the volume, distribution and balance of topics, opinions and authorship within medicine’s literature [1–3]. At the level of article *content*, commercial considerations may affect the composition of articles, the design, data coding, analysis and interpretation of the studies that inform them, the findings they highlight, the opinions they express and the language they use [1–13]. Such influence may be exerted through numerous channels, including company design of trials, analysis, interpretation and ownership of trials data, the use of hired contractors, the use of company coauthors, the use of commercial editorial teams to develop manuscripts and the selection of accommodating academics to work on industry projects.

**Table 1:** Potential commercial bias in medical journal articles, and ICMJE protection

Level of bias (References)	Examples	Current ICMJE protection (section of December 2015 *Recommendations*)
Academic discourse and commercial planning [1–3]	• Clinical trials: what studies to undertake, whom to recruit as partners, whether and how to publish. • Publications: number, theme, placement, scheduling and authorship of articles.	• Limited options available to ICMJE. • Clinical trial registration (III L). • Duplicate/prior publications (III D).
Article content [1–13]	• Clinical trials: design, conduct, discontinuation, analysis and interpretation. • Data misrepresentation, fabrication and falsification. • Review criteria and literature search. • Insertion of commercial “key messages.” • Language and rhetoric.	• Limited options– heavily reliant on peer review. • Contributor listings (IIA 1). • Stringent author accountability (IIA2). • Access to data (IIB, IIB 2). • Authors’ right to publish. • Reporting guidelines (IV A2). • Commercial supplements and series – journal’s editorial control maintained (IIIG). • Rules on correction or retraction of inaccurate or fraudulent data (IIIB).
Article attribution [1, 2, 13–15]	• Spinning the sum effect of authorship, contributorship, acknowledgements, text, labeling, etc. • Depends on what readers perceive, not merely what is disclosed. • Advocacy-based marketing foregrounds recruited academic authors while obscuring the proprietary role of commerce using small print, vague language and omission.	• Extensive options – but poorly developed. • Ban on ghost authorship (II A2). • Funding source, trial identifier listed visibly in Abstract (IV A3). • Contributorship, interest declarations and “Sources of support” sections provide limited benefits but involve small print (IIA, IIB, IV A3). • Declaration of product being marketed – but only for supplements (IIIG).

Finally, with respect to the *attribution* of commercial articles, the identities, roles and interests of contributors and stakeholders may be communicated to readers in a misleading way. In particular, the role of companies may be downplayed and that of academic recruits exaggerated, for instance by placing academics at the head of the author byline and crediting companies only in minor author placements and fine print [1, 2, 13–15]. Such abuses of attribution are the basis of “advocacy”-based marketing, in which, for reasons of endorsement, commercially-instigated data and argument are presented to customers by their own respected peers rather than companies themselves [2, 15–18].

The International Committee of Medical Journal Editors (ICMJE) is preeminent in setting editorial standards for medical journals. Its *Recommendations* – previously known as the *Uniform Requirements for Manuscripts* (*URM*) [19] – have gradually evolved since their debut in 1978, and currently set standards on authorship, conflict of interest, submission and review, publishing and editorial issues, and manuscript preparation. They have been adopted by nearly 2000 journals including most leading titles, although ICMJE membership is limited to a handful of top journals, some more minor and non-English language journals, the US National Library of Medicine and the World Association of Medical Editors. Several past and present ICMJE editors have taken principled public stands against commercialization within medicine, [5, 20, 21] and the current *Recommendations* include measures that help check commercial bias on all three levels I have described. Yet the *Recommendations* are also embraced by industry, including trade associations of the publishing, writing, marketing and pharmaceutical sectors [22–25] and this raises the question of whether the ICMJE’s existing measures to address the problem of industry bias are adequate, or whether further steps are required. In 2011, I and others showed how the then-ICMJE authorship guidance was in fact commodious to marketing, [14, 26] prompting revision by the ICMJE. Here, I have examined the entire *Recommendations* rather than the authorship formula alone, evaluating every section in respect of my direct working knowledge of industry practices. I identify numerous protections against commercial distortion, but also widespread weaknesses that facilitate marketing practices. I argue that these weaknesses derive not only from ignorance about marketing, but from longstanding conceptual shortcomings within editorial thought and publication ethics.

## Discussion

ICMJE measures to address bias at the level of discourse, content and attribution are summarized in Table 1. The strengths and weaknesses of successive sections of the *Recommendations* are summarized in Table 2.

**Table 2:** Section by section: strengths and weaknesses of the ICMJE *Recommendations* as a barrier to marketing bias (December 2015 Recommendations)

Section		Strengths	Weaknesses
IIA1	Contributor listings	• Bookkeeping of author accountability. • Helps enforce integrity of publication.	• Do not separate academic and company coauthors. • Small print, vague language assist attribution bias.
II A2	Authorship formula	• Encourages high scientific standards. • Encourages rigorous checking by authors. • Bans guest authorship.	• Facilitates byline avoidance. • No advice on author order. • No company-level authorship or accountability.
II A3	Acknowledgements	• Bookkeeping of non-author contributions, accountability.	• Omit key commercial information. • Small print, vague language assist attribution bias.
II A3	Writers barred from coauthorship	• None.	• Supports commercial production of ghostwritten manuscripts. • Facilitates and downplays commercial content influence.
II B	Conflict of interest	• Comprehensive net for author financial interests.	• Salient interests not highlighted. • No contributor interests. • No corporate interests.
II B	Role of funding source	• Company role in conducting research, decision to publish reported.	• No requirement to report company instigation, database ownership or identity of marketed product.
II B	Access to data	• Discourages restrictions on author access to data.	• No requirement for independent statistical analysis. • No demand for contractual rights to data access and unrestricted usage.
IIB	Right to publish	• Authors discouraged from agreeing limitations to their right to publish.	• Should insist on contractual rights guaranteeing publishing rights for academic coauthors.
IIID	Overlapping publications	• Reduces discourse bias by identifying / reducing duplicate articles.	• Limited ability to prevent repetition of commercial content.
IIIG	Supplements and series	• Editorial control defended. • Notification of product being marketed.	• None – but should apply to all commercially financed articles.
III L	Trial registration	• Helps locate published commercial trials. • Helps expose non- or inadequate publication.	• None.
IV A2	Reporting guidelines	• CONSORT compliance required. • Minimal methodological standards supported.	• Readers referred to EQUATOR and National Library of Medicine (NLM) listings of guidelines - these include trade guidelines compatible with marketing.
IV A3b	Funding source, trial identifier listed in Abstract	• Visibly identifies presence of company in PubMed-searchable format.	• The terms “funding,” “support” and “sponsorship” misrepresent actual instigating/proprietary role of commerce.
IV A3d	Role of “contracted organization” described in Methods	• Reveals involvement of company to readers. • Also identifies contract research organizations.	• Stipulation is vaguely worded and omits key details e.g. company instigation, database ownership, identity of marketed product.

### Discourse-level bias

The ICMJE’s options to prevent discourse-level bias are limited, since the *Recommendations* are largely concerned with individual submissions to journals rather than the research and planning that precedes them. Nonetheless, several measures have been introduced, including limited measures on prior and repeat publication, practices which might be used by marketing to increase visibility of a commercial product, and most importantly, trial registration. Since 2005, the ICMJE has insisted that trials are registered at or before patient enrollment begins, at a major public registry; this measure was specifically designed to address the problem of selective reporting of trials [27]. The United States Food and Drug Administration Amendment Act of 2007 has since made trial registration a legal requirement, requiring the results to be deposited in a publicly available database [28]. It is important to recognize the role played by the ICMJE in driving these improvements in public knowledge about industry research.

### Content-level bias

It is largely for peer reviewers to identify content bias, although the ability of peer review to cope with the volume and technicality of industry material has been questioned [5]. The ICMJE does, however, place rigorous accountability upon authors, as discussed below in respect of authorship, and demands adherence to the Consolidated Standards of Reporting Trials (CONSORT) [29]. Together, trial registration and CONSORT adherence should ensure that all prospectively defined endpoints are reported in ICMJE-compliant journal articles – although these measures cannot control how results are framed, interpreted and spun.

#### Data ownership, analysis and access

A longstanding area of weakness for the ICMJE concerns company clinical trials data. The ICMJE does not require independent data analysis, or the academic authors to have possession of, or to have looked at, the full data themselves. All that is required is that they should be granted “access” to the data if they request it. In practice, many academic authors only see a results summary prepared by the company. Section IIB of the *Recommendations* advises authors to “avoid entering in to agreements” with companies that might limit their access to the basic data, but this merely advises against restrictive agreements, whereas it should insist on contracts requiring senior academic authors to have full, immediate and enduring use within their institutions of the anonymized patient-level database, with unrestricted rights of analysis, comparison and dissemination.

Concerns about the meaning of “access to data” and similar proposals to these have been discussed by a number of scholars [30–33]. Accordingly, the most important current development in the ICMJE *Recommendations* is the impending introduction of guidance on data sharing [34]. Pharmaceutical companies have long resisted making trials data publicly available, chiefly on the grounds that other companies might mine it for their own drug development programs, although these arguments have been criticized [32, 33]. A greater problem would likely be data exploitation by competitors for their own products’ commercial benefit. This could lead to escalating scientific misinformation, negative spin and fatuous research as companies with competing products mined one another’s data to joust for advantage.

There is therefore a plausible case for preventing companies from accessing other companies’ patient-level data, although protocols, summary results and clinical study reports should be publicly available for all trials. Independent academic groups should, however, have full, retrospective access to clinical record forms and patient-level data, including unpublished trials, for any drug with at least one approved indication in any market [32, 33]. Furthermore, any form of data analysis whatsoever should be permitted, including head-to-head comparisons with competitors and pooled analyses across trials from different manufacturers. New organizations independent of companies and researchers would be necessary to manage these arrangements.

It is unlikely the initial ICMJE guidance on data sharing will approach these standards. The draft ICMJE proposals released in January 2016 suggested requiring data sharing only in relation to results reported in the published article, rather than full study databases, and made no mention of protocols, clinical study reports or clinical record forms [34]. Furthermore, the proposals indicated potential ICMJE willingness to accept, and help enforce, data sharing agreements restricting what analyses would be permissible. Therefore, while the introduction of an ICMJE data sharing policy is welcome, it may prove unduly accommodating of commercial restrictions – an outcome which could establish a barrier, not a gate, to proper data sharing. One positive feature of the draft proposals was a requirement for the data sharing plan to be included as a component of public trial registration.

#### Manuscript development

An important area of weakness for the ICMJE concerns manuscript development, which may be a source of content bias. Firstly, academics performing key roles in industry projects including drafting are more likely to have financial ties to industry than their academic coauthors [35]. A second means of potential influence is the use of employees as coauthors – they may be heavily involved in manuscript development, and serve as a conduit for input from unnamed colleagues, yet receive only inconspicuous credit in the middle of the byline.

The ICMJE offers no guidance on employee coauthors, but does address a third potential conduit for commercial influence on manuscript development, the use of trade writers. Many academics in other fields would consider the use of trade writers to draft learned text an affront to scholarship, but medical journals and the ICMJE have embraced the practice, on the basis that writing is supposedly a technical, not an intellectual task. Perhaps the most signal exchange occurred in 1995 in JAMA, which though 20 years old merits quotation [36]. DeBakey and DeBakey defended the traditional scholarly perspective that writing is an intellectual task, such that trade writers should have no place in learned literature:

There is a clear distinction between minor editing and ghostwriting; the first involves inconsiderable changes, the second composing… Writing a competent medical report requires thinking logically, analyzing data rigorously and interpreting them critically, organizing relevant material coherently, and presenting results in a lucid form. Writing, if done thoughtfully, may disclose an ill-defined thesis, experimental flaw, or faulty data analysis. How many ghostwriters apply such scrutiny? How can the reader judge the scientific merit of a ghostwritten article? It may be eloquent and persuasive without being true or valid….. Instead of bringing ghostwriters out of the closet, let's flush them out of the profession.… Let's exorcize the ghosts, not acknowledge them.

But JAMA editors Flanagin and Rennie took the contrasting view that trade writing is mere “literary assistance” for inarticulate doctors, and therefore acceptable:

… a writer, who solely contributes to the writing of a manuscript – visibly or not – does not automatically qualify for authorship…. such assistance should be disclosed, not prohibited. By acknowledging substantial writing and editing assistance, we are not judging whether such aid biases a report… We know of many scientists who require such assistance. … So why shouldn't the actual drafter of the manuscript be publicly credited for such a contribution in the acknowledgment, especially if the writer is employed or paid by a company with a commercial interest in the report? … crediting literary assistance is thoroughly compatible with our efforts to preserve the integrity of scientific publication.

Today’s ICMJE policy follows the JAMA view; yet the DeBakeys’ view of writing as composition was correct, and its rejection has facilitated commercialization of academic medical literature. What is most lacking in the JAMA-ICMJE viewpoint is an understanding that while company editorial teams may not have a true expert’s knowledge of the *medical* subject matter of the articles they draft (though most trade writers have science PhDs), theirs is an intellectual contribution to content nonetheless, and their expertise often *exceeds* that of the academic authors in two respects - the intellectual task of manuscript composition, and more importantly, their awareness of the commercial nuances of the text. Furthermore, their work is reviewed by company personnel who include true subject-matter experts. In privileging the expertise of academic authors and treating other contributions as auxiliary, medicine’s editorial community has played into the hands of marketing, blinkering itself to the ease with which highly intelligent parties, who for marketing reasons wish to be perceived as mere “assistants”, can subtly frame and spin the content of literature in the service of commerce.

### Attribution and disclosure

Arguably the greatest inadequacies of the current *Recommendations* concern the attribution of journal articles. The word “attribution” is not currently used in the *Recommendations*, and it is important for the editorial and ethics communities to consider carefully what attribution involves and how it can be subjected to systematic commercial spin.

Attribution is not coextensive with authorship. Correctly understood, the attribution of a journal article encompasses everything it communicates in order to inform readers about its provenance and development, the identity of its stakeholders and contributors, and their roles and interests. Attribution involves authorship; author sequence; contributor listings; statements in the abstract; and sometimes information provided in the title, footnotes or labeling. Interest declarations also have a bearing upon attribution since they point to the motivations at play in the work. It is also very important to note that attribution is not merely a matter of *documentation*, but of *communication with the reader*. Accordingly, the parties that played the most important role in developing the article and conducting the research it describes should be represented most prominently.

Unfortunately, the attribution of industry literature is frequently subjected to commercial bias in the service of “advocacy”-based marketing. As discussed above, academic authors are commonly highlighted, while the role of the company is downplayed through minor author placements, vague disclosures and small print [14, 15]. The ICMJE *Recommendations* support or tolerate these unethical practices.

#### Authorship

Authorship is the most important component of attribution within scholarly literature, and has a privileged standing within medicine. The ICMJE authorship formula is highly restrictive, and currently has four conditions:

1. Substantial contributions to the conception or design of the work; or the acquisition, analysis, or interpretation of data for the work; AND
2. Drafting the work or revising it critically for important intellectual content; AND
3. Final approval of the version to be published; AND
4. Agreement to be accountable for all aspects of the work in ensuring that questions related to the accuracy or integrity of any part of the work are appropriately investigated and resolved.

This formula is designed to ensure that all authors were truly involved in the work, and are willing and able to account for it. Its stringency reflects the need for a consistent mechanism to regulate authorship on projects with numerous participants, but it also reflects the shock suffered by academic medical publishing in the 1980s and 1990s, when fraudulent literature was exposed and personal research integrity became an overriding ethical concern [37, 38]. Accordingly, the ICMJE insists that authors must have done the research (Condition 1), worked on the manuscript (Condition 2) and approved the published text (Condition 3). The fourth condition seeks to reconcile author accountability with varying author expertise. Most clinicians cannot, for instance, be technically responsible for an article’s statistics, or statisticians for clinical interpretation, but both must ensure issues arising in either domain are properly addressed.

With respect to commerce, the effects of the ICMJE authorship formula are mixed. Creditably, it militates against lax scientific standards, compels authors to review research and text rigorously, and opposes “guest authorship” – that is, inclusion of authors whose contributions are too modest to merit author status. These benefits apply in commercial and noncommercial work alike. There are, however, four aspects of the ICMJE approach that prove commodious to marketing. Firstly, there is no facility for corporate authorship. This reflects the longstanding preoccupation of medicine’s editors with personal accountability, illustrated by Rennie’s sentiment that “attached to every article there be actual beings, prominently identified, who are prepared to be accountable for all aspects of the reports” [39]. Yet in commercial publications, the company itself usually plays a highly active role in the conception, design, conduct and analysis of research, and in planning and drafting manuscripts. Typically this involves numerous individuals, both within the company itself and in its hired agencies [2, 3, 14]. Many of these individuals do not qualify as authors in their own right by the ICMJE formula, or indeed as non-author contributors. Corporate authorship is a reality of industry science, but in focusing with such stringency on individuals rather than requiring companies themselves to be credited with an authorial or similarly prominent attribution, the current ICMJE guidance facilitates *de facto* corporate ghost authorship.

Secondly, there is no adequate ICMJE guidance on author sequence. In particular, there is no injunction against the “academic frontloading” of author bylines for the purposes of credibility [14, 15]. So ingrained and expected has academic frontloading become that many companies now view it as a necessity for maximizing the likelihood their work will be accepted by journals for publication [40, 41].

Thirdly, the ICMJE takes the remarkable stance that no medical journal article need ever be written by any of its named authors. Clause 2 of the authorship formula states that revision alone is required; and further text below the formula bars what is ambiguously termed “writing assistance” from byline credit. Consequently, a significant intellectual and commercial contribution to the published work is credited only in fine print. The *Recommendations* thereby sanction the industrial production of content using ghostwriting – “where a professional medical writer prepares a manuscript on behalf of a named author, but the writer is not listed as an author” [42]. The ICMJE should replace this discreditable policy with the approach exemplified by *Neurology,* and require writers to be credited as authors [43].

Fourthly, the *Recommendations* do not specifically *require* key contributors to be authors. The ICMJE formula was conceived as a means of limiting authorship, and does not address the possibility that parties who should be authors might prefer not to be – notably, commercial parties seeking to direct reader attention to academic authors for purposes of endorsement. Text introduced in 2013 states that individuals substantially involved in the work and therefore meeting Condition 1 “should have the opportunity” to work on the manuscript and meet Conditions 2–4, therefore becoming authors. Yet there is no specific demand they should do so, and companies have been known to reduce the number of their deserving employees who become coauthors in order to reduce company visibility [40]. A more robust approach would be to require all those substantially involved in research to be compulsorily identified as contributors, and a written explanation provided to editors when individuals so named chose not to participate as authors.

#### Contributorship

The ICMJE does not formally recommend contributor listings, but “strongly encourages” them. Contributor listings benefit noncommercial and commercial publications alike, permitting a more granular account of credit and responsibility, and providing a record should concerns about misconduct arise [37]. In industry literature, contributor listings have the additional advantage of helping identify the roles played by industry coauthors.

Currently, contributor listings vary considerably among journals. The clearest and most informative format is exemplified by *JAMA,* which uses structured endnotes organized by contribution type to record the roles of both authors and non-authors. Currently, the CRediT (Contributor Roles Taxonomy) program, a Consortia Advancing Standards in Research Administration Information (CASRAI) initiative, [44, 45] is attempting to develop a standardized approach similar to JAMA’s but involving 14 distinct categories of contribution. This approach is likely to gain support from journals and in due course the ICMJE. The fourteen categories are broadly reasonable, although certain key industry roles such as instigation, finance, data ownership and product marketing are absent. The success of the system in respect of industry will depend on whether these omissions are addressed and whether companies, as well as individuals, are to be systematically credited.

But while contributor listings are important, they also illustrate the failure of medicine to distinguish adequately between documentation and communication. The former requires only fine-print; the latter is thwarted by it. Rennie and colleagues’ 1997 proposals suggested contributorship should replace authorship; but they also argued for retention of the byline, for “those who contributed most substantially to the work” [46]. This amounts to a *de facto* retention of authorship, in contrast Smith’s call for authorship’s genuine replacement by a “film credits” approach [47, 48]. Furthermore, Rennie and colleagues called for the contributions themselves, including the names of non-byline contributors, to be documented in fine print. Alternative proposals by Fotion and Resnick sought to establish more conspicuous credit for contributors who were not byline authors, avoiding what Fotion derided as “the almost meaningless” option of crediting them in a footnote [49, 50]. The ICMJE has broadly followed Rennie’s proposals, save that those privileged with byline status continue to be termed and indexed as authors. While the documentary function of contributor listings is an important advance, it therefore provides marketing with a fine print format wherein the role of the company can be mapped to inconspicuous employees, while recruited academic authors continue to dominate reader perceptions.

#### A critical weakness: reader notification and labeling

The weaknesses of the *Recommendations* with respect to authorship and contributorship would in large measure be negated if there was also guidance on prominent labeling of industry publications, requiring readers to be notified at the outset of an industry article’s true provenance, ownership and commercial functions. This could be achieved through a variety of means, for instance naming the company responsible for the work as a lead author, or in the title, or in a label at the head of the text. Such notification would need to be conspicuous, clearly worded, and apparent when the article abstract was viewed online. Clear commercial labelling would also combat the use of academics for endorsement and the production of scholarly articles as marketing vehicles.

Unfortunately, the *Recommendations* contain no provisions for prominent commercial labeling, except in certain non-standard publication settings (Table 2). Most usefully, section IVA states that “Funding sources should be listed separately after the Abstract”, and some journals now credit companies with “funding” or “support” at this location. Yet while this is commendable, the language is misleading and euphemistic, because pharmaceutical companies are not mere sources of “funding”. Indeed, it is notable throughout the *Recommendations*, that oblique language is used when referring to industry. The words “company”, “business”, “corporation”, “pharmaceutical” and “marketing” are entirely absent, and the terms “sponsor” and provider of “support” are preferred. This helps sustain a false depiction of industry research, in which the article and the study it reports appear to belong fundamentally to the authors, while the company that is in fact responsible for both and which owns the data appears to have a secondary and subservient status.

### Conflict of interest reporting

The ICMJE publishes a detailed conflict-of-interest form which has helped standardize author interest disclosures. The importance of author interest disclosures has recently been debated within the editorial community and the standards set by the *Recommendations* have been defended [51, 52]. Notwithstanding their importance, however, the ICMJE disclosure guidance has limitations in respect of commercial projects. Firstly, only authors are required to disclose interests. Other contributors are not, and if the byline is evaded, no disclosure is required. Secondly, the confusion between documentation and communication is once again evident, in that: there is no provision for bringing the most salient interests – namely, authors’ relationships with the company marketing the drug – to the attention of readers. Consequently, these relationships are often rendered inconspicuous by being reported amid a mass of other disclosures, and in small print. Finally, there is no requirement for disclosure of other stakeholder interests – most importantly, company interests such as the identity of the drug being marketed in the article. Journals too are stakeholders whose interests should be reported [53–55].

Interest disclosures present a challenge for print journals because of space considerations; some journals refer readers to online author disclosures. Such problems will increase if articles are required to list more extensive interests including those of stakeholders and non-author contributors. Three steps appear necessary to address these difficulties: firstly, to ensure that critical interests, such as company finance and authors’ relationships with the company, are brought conspicuously to readers’ attention at the outset; secondly, to accompany articles online with standardized grids documenting the interests of companies, authors, contributors and the journal; and thirdly, to establish public registries of comprehensive author interests, as advocated by Rasmussen and colleagues [56]. Such registries might be the responsibility of the state, academic institutions or potentially author identification systems such as ORCID [57]. All these possibilities require discussion and debate, but as with trial registration and data sharing, interest registration provides an opportunity for the ICMJE to constructively influence research culture.

### Factors underlying ICMJE commercial policy weakness

It is clear that without the *Recommendations*, the quality of medical literature, including commercial medical literature, would be significantly poorer than it is today. Equally, however, the marketing of blockbuster drugs involves industry-financed literature that is commercially biased but ICMJE-compliant. The ICMJE accepts arrangements in which companies instigate trials, analyze and own the data, and plan and produce manuscripts, but attribute these projects primarily to recruited academics. These academics are not guest authors insofar as they contribute actively to projects, and yet they are contingent: were they not available, other academics could and would be recruited, and publications with similar content and identical commercial functions would be generated. The ICMJE *Recommendations* facilitate these practices, and showcase in their own text a euphemistic language of “sponsors”, “funding” and “support” which falsely implies the corporate masters of this research are mere facilitators.

These weaknesses in the ICMJE *Recommendations* are of long standing, and likely reflect a diversity of underlying causes. Most obviously, it might be considered inevitable that medical publishing’s financial dependency on the pharmaceutical industry should lead to timid editorial standards and incomplete measures. But against such considerations must be set the principled record of some ICMJE editors in resisting commercial influence, and the organization’s substantial achievements, for instance on trial registration.

In fact, a welter of factors probably contribute to the weaknesses in ICMJE policy. These likely include editors’ historical preoccupation with the problem of author accountability, which while important has reduced consideration of other issues; inadequate understanding of commercial practices; the assiduous public relations maneuverings of the publications-marketing trade; the cultural haughtiness of medicine, whose emphasis on professional qualification, status and authority leads naturally to the denigration of writers and elevation of key opinion leaders; the cultural insecurity of academic medicine, tacitly compliant with corporate science but clinging to the eidolon of its own lost sovereignty; and a conjunction of self-interest among the pharmaceutical and marketing industries, publishing houses, journals, editors and academic authors, whose finances or careers all benefit from the prevailing editorial and marketing culture.

Finally there are conceptual shortcomings within editorial thought, among which the most interesting is perhaps the issue of attribution. Medicine’s editors must understand that attribution is multi-faceted and that even if an article meticulously and fully discloses the role of all its contributors and stakeholders – and this is rarely the case in contemporary industry literature – it will remain poorly attributed if the most prominent features of its attribution do not identify to the reader the dominant parties in its provenance and development. The publication ethics community too has been preoccupied with the important issues of documentation and disclosure to such a degree that it has largely failed to address the importance of communication with the reader. The marketing trade continues to profit from an understanding of attribution subtler than that of editors and ethicists alike.

### Suggested developments of the *Recommendations*

Table 3 summarizes for discussion some areas of potential improvement for the *Recommendations* in respect of industry. In addition to these, the ICMJE should work alongside academic institutions and societies to achieve an outright ban on advocacy marketing – defined as the dominant attribution of commercially-owned, instigated or financed projects to academics and their institutions. It is reasonable for academics to work with industry in the clinical evaluation of commercial drugs, and to participate in reporting the findings to their medical colleagues; but such projects should always be presented as commercial ones with academic involvement, not the other way round.

**Table 3:** Future development of the ICMJE *Recommendations*: suggested policies with respect to industry

Theme	Policies for discussion
General commercial guidance	• Develop specific section on commercial publications. • Encourage CONSORT to develop a specific commercial schedule. • Do not refer users to self-interested trade guidelines [22–24]. • Initiative to ban advocacy-based marketing in scholarly publications.
Data sharing	• Full rights of access to study protocols, clinical study reports, deidentified patient data including clinical record forms. • Unrestricted freedom of data analysis, comparison and pooling. • Database access/analysis rights limited to bona fide academic research groups.
Attribution	• Guidance on balanced attribution, ensuring conspicuity of lead contributors – including companies as corporate entities. • Guidance on author sequence, corporate authorship. • Cease ICMJE support for ghostwriting – all major contributors to studies/manuscripts, including writers, required to be coauthors. • Up-front reader notification of commercial projects (e.g. company named in title or as a lead author). • Role-based contributor listings, including company roles (instigation, finance, product marketing, data ownership). • Cross-media standard on attribution/labeling. • New NLM publication type – “Commercial”. • Work with NLM, CRediT initiative to develop new PubMed-listed categories of credit alongside author – including categories suitable for companies.
Interest disclosures	• Financing company’s interests and author relationships identified in Abstract. • Online grid of all stakeholder interests including authors, contributors, companies and journal. • Initiative to establish public repositories for comprehensive author interest disclosures.

Furthermore, the ICMJE should apply the principles of disclosure it requires of authors to itself. No interest disclosures are currently to be found on the ICMJE website or in the *Recommendations*, nor is there information on how the ICMJE *Recommendations,* secretariat, meetings and other activities are organized and financed. This is unacceptable – especially since some ICMJE member journals, who pay the salaries of editors who decide the ICMJE rules, belong to commercial publishing houses, profit from reprint or advertising sales to industry, or are stocked with industry manuscripts.

## Conclusions

The ICMJE *Recommendations* are limited in their capacity to prevent commercial bias, but provide valuable protection against the worst potential commercial abuses of literature, such as low methodological standards, guest authorship and nondisclosure of industry involvement. The *Recommendations* have also been used to raise research standards, for instance in trial registration and the emerging guidance on data sharing. Unfortunately, much of the ICMJE guidance with respect to industry remains weak and incomplete, and advocacy-based marketing is directly facilitated. The ICMJE must bear a degree of responsibility for deficiencies in the quality of medical literature, to the extent that these failings are overlooked or even succored by the standards the ICMJE applies. One must however conclude by supporting the ICMJE *Recommendations* as a work continually in progress, by acknowledging their many beneficial effects on research and publication standards including in commercial literature, by encouraging their continuing evolution, and by asserting the value of these flawed but essential guidelines.

### Ethics approval and consent to participate

Not applicable.

### Consent for publication

Not Applicable.

### Availability of data and materials

The ICMJE *Recommendations*, website and editorials are available online at www.icmje.org.

## Abbreviations

ICMJE: International Committee of Medical Journal Editors
CONSORT: Consolidated Standards of Reporting Trials
